# Diffractive imaging of a rotational wavepacket in nitrogen molecules with femtosecond megaelectronvolt electron pulses

**DOI:** 10.1038/ncomms11232

**Published:** 2016-04-05

**Authors:** Jie Yang, Markus Guehr, Theodore Vecchione, Matthew S. Robinson, Renkai Li, Nick Hartmann, Xiaozhe Shen, Ryan Coffee, Jeff Corbett, Alan Fry, Kelly Gaffney, Tais Gorkhover, Carsten Hast, Keith Jobe, Igor Makasyuk, Alexander Reid, Joseph Robinson, Sharon Vetter, Fenglin Wang, Stephen Weathersby, Charles Yoneda, Martin Centurion, Xijie Wang

**Affiliations:** 1 Department of Physics and Astronomy, University of Nebraska-Lincoln, 855 N 16th Street, Lincoln, Nebraska 68588, USA; 2 PULSE Institute, SLAC National Accelerator Laboratory, Menlo Park, California 94025, USA; 3 Institute of Physics and Astronomy, Potsdam University, Potsdam 14476, Germany; 4 SLAC National Accelerator Laboratory, Menlo Park, California 94025, USA

## Abstract

Imaging changes in molecular geometries on their natural femtosecond timescale with sub-Angström spatial precision is one of the critical challenges in the chemical sciences, as the nuclear geometry changes determine the molecular reactivity. For photoexcited molecules, the nuclear dynamics determine the photoenergy conversion path and efficiency. Here we report a gas-phase electron diffraction experiment using megaelectronvolt (MeV) electrons, where we captured the rotational wavepacket dynamics of nonadiabatically laser-aligned nitrogen molecules. We achieved a combination of 100 fs root-mean-squared temporal resolution and sub-Angstrom (0.76 Å) spatial resolution that makes it possible to resolve the position of the nuclei within the molecule. In addition, the diffraction patterns reveal the angular distribution of the molecules, which changes from prolate (aligned) to oblate (anti-aligned) in 300 fs. Our results demonstrate a significant and promising step towards making atomically resolved movies of molecular reactions.

## Abstract (web summary)

Imaging changes in molecular geometries with sufficient temporal and spatial resolution to image nuclei is a critical challenge in the chemical sciences. Here the authors report gasphase Megaelectronvolt electron diffraction with 100 fs temporal resolution and subAngstrom spatial resolution.

## Graphical abstract

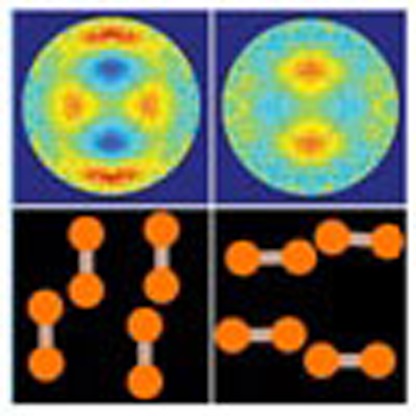

The dynamical behaviour of molecules is governed by the complex interplay of a correlated system of nuclei and electrons which interact via Coulomb and exchange forces. Determining how the individual nuclei within a molecule move relative to one another during a molecular transformation represents a key step to understanding chemical reactivity. Developments in the field of femtochemistry enabled capturing the motion of nuclear wavepackets using purely spectroscopic measurements with femtosecond laser pulses^1^,^2^,^3^,^4^,^5^,^6^,^7^,^8^. For small molecules, structural information on the nuclear geometry can be indirectly inferred based on exact knowledge of spectral transitions involved in the probe process. For larger molecules, this inference becomes unfeasible. Diffraction techniques, which provide direct access to the position of each atom within a molecule, are a more effective approach. Over the last few decades, great strides have been taken to observe ultrafast dynamics with ultrashort X-ray pulses from synchrotrons and free electron lasers (FELs)^9^,^10^,^11^,^12^ and via diffraction of short electron pulses^13^,^14^,^15^,^16^. With the advent of FELs, X-ray diffraction experiments have now reached a sub-100 fs temporal resolution. The spatial resolution is sufficient to observe larger-scale molecular processes like light-induced opening of a six-membered ring, but not to resolve individual atoms because of the limited wavelength available in FELs^12^. Laser-induced electron diffraction^17^,^18^ and photoelectron holography^19^ can also provide spatial and temporal resolution simultaneously, but require significant theoretical input to retrieve the interatomic distances. Ultrafast electron diffraction (UED) from gas-phase molecules has achieved sub-Angstrom spatial resolution but the temporal resolution has not been sufficient to observe molecular geometry changes on the femtosecond timescale^20^,^21^. Here we report a crucial advance in gas-phase UED using multi-MeV relativistic electron pulses to achieve a temporal resolution of 100 fs root-mean-squared (RMS), or 230 fs full-width at half-maximum (FWHM) that opens the door to observing the motion of individual nuclei that result from structural changes during photochemical reactions of isolated molecules.

The majority of UED experiments have been performed on solid-state samples^22^,^23^, which are ideal to understand collective effects in condensed media such as superconductivity, heat transport and magnetism. Gaseous molecules are ideal to study prototypical processes in chemistry. They also provide a direct link between experiments and quantum chemical calculations, which can be performed on the highest level for isolated molecules^23^,^24^,^25^. Early gas phase UED studies employed stroboscopic electron diffraction^26^. A breakthrough was achieved when picosecond electron beams became available in late 1980s (ref. 27). Zewail and co-workers were able to resolve non-equilibrium molecular structures^28^, transient molecular structures^15^ and radiationless dark structures^16^ using picosecond UED. However, in order to resolve molecular geometry changes in real time, a better temporal resolution is required. In the context of the crucial excited state photoisomerization reactions^24^, a 200-fs resolution is suitable for exploring the nuclear dynamics of the isolated azobenzene isomerization^29^. In addition, this temporal resolution is sufficient to explore photoprotection processes of nucleobases^30^,^31^,^32^,^33^.

To improve UED temporal resolution, two major effects must be overcome: space-charge repulsion between electrons^34^ and velocity mismatch^35^ resulting from the electron pulse lagging behind light pulses used for the molecular excitation. Both of these limitations can be minimized using relativistic MeV electrons^36^,^37^,^38^,^39^,^40^,^41^,^42^,^43^,^44^,^45^. The longitudinal space-charge pulse elongation is proportional to 1/*β*^2^*γ*^5^, where *β*=*v*/*c*, *γ*=(1−*β*^2^)^−1/2^ is the Lorentz factor, and *v* and *c* are the speed of electrons and the speed of light in vacuum, respectively^46^. Concerning the velocity mismatch, electrons with 3.7 MeV kinetic energy travels at *v*=0.993*c*. This results in only 5 fs delay with respect to an optical pulse for a typical 200 μm interaction length.

Here we present the ultrafast laser-induced rotational dynamics of N_2_ molecules in the gas phase. We impulsively excited a rotational wavepacket in a N_2_ gas sample using a 35-fs FWHM laser pulse, spectrally centred at 800 nm, which interacts non-resonantly with the anisotropic molecular polarizability tensor. In the impulsive alignment regime, the alignment laser pulse duration is much shorter than the rotational period of the molecule, and the ensemble reaches its maximum degree of alignment after the interaction with the laser pulse. The phase evolution of the rotational wavepacket results in rotational revivals, with molecules alternating between aligned and anti-aligned directions^47^,^48^. Previous efforts to investigate rotational wavepackets with UED have captured dynamics on picosecond timescales^49^,^50^. The rotational dynamics of laser-aligned N_2_ has been previously observed with optical birefringence^51^, strong field ionization^52^, high harmonic generation^53^,^54^ and Auger electron spectroscopy^55^. We have observed the temporal evolution of the full wavepacket revival with an 8.35 ps period by quantifying the anisotropy in the diffraction patterns. The temporal resolution was determined by a fitting routine using the measured dynamics. Furthermore, we have retrieved molecular images of the aligned and anti-aligned molecular ensemble with atomic resolution.

## Results

### Experimental layout and static diffraction

**Figure 1:**
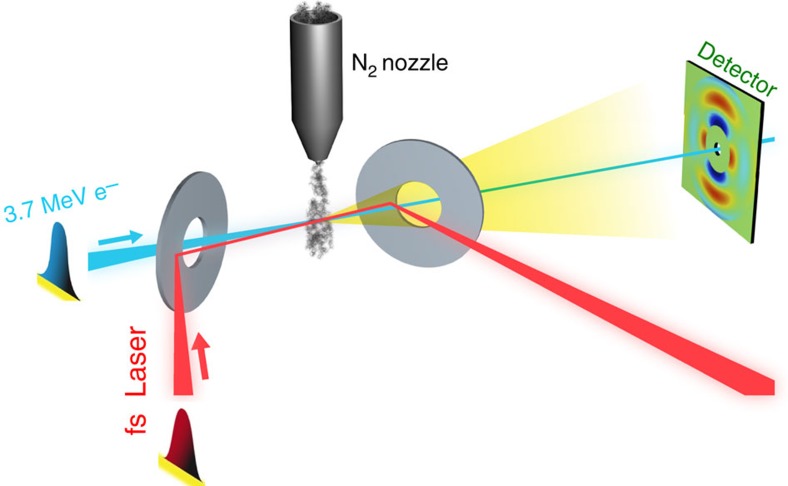
Experimental layout. A sketch of the experimental setup. A 3.7-MeV pulsed electron beam (blue) is directed towards a nitrogen gas jet (grey). The gas jet is introduced into the vacuum chamber using a pulsed nozzle (black). The pump laser pulse (red) is deflected by two ring-shaped mirrors. The laser propagates at a small angle (∼5°) with respect to the electron beam as it traverses the target, and is then deflected away from the detector by the second mirror. The electron diffraction pattern is recorded with a phosphor screen located 3.1 m downstream from the interaction region. The unscattered electron beam is transmitted through a hole in the phosphor screen.

The experimental layout is shown in Fig. 1. The electron pulse (blue) is diffracted from the nitrogen gas jet (grey), which is introduced into the vacuum chamber using a pulsed nozzle (black). The diffraction pattern is captured by a phosphor screen and a detector. The 800-nm alignment laser pulse (red) is directed to the target and removed from the vacuum chamber by two holey mirrors at a 45° angle to the electron beam. The full setup is discussed in detail elsewhere^56^. The electron and the laser beam have a small angle of ∼5°. This makes the velocity of the laser 0.996c along the electron beam direction, very close to 0.993c, the velocity of the electron beam. Therefore, the velocity mismatch is nearly eliminated. More details of the experiment can be found in the Experimental Setup section in the Methods.

The diffraction pattern is commonly expressed as a function of the momentum transfer


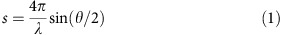


where *λ* is the wavelength of the electron beam and *θ* is the angle between the scattered and transmitted electrons. For a 3.7-MeV electron beam, *λ*=0.30 pm. The total scattering intensity *I*_tot_ is the sum of the atomic scattering intensity *I*_at_ and the molecular scattering intensity *I*_mol_. *I*_at_ is defined as


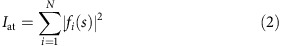


where *N* is the number of atoms in the molecule and *f*_*i*_ is the elastic scattering amplitude for the *i*th atom. For MeV electrons, *f*_*i*_ can be calculated using the ELSEPA program^57^.

The structural information of the molecule is encoded in the molecular scattering intensity, *I*_mol_, given by


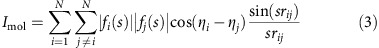


where *η*_*i*_ is the scattering phase of the *i*th atom and *r*_*ij*_ is the distance between the *i*th and the *j*th atoms^20^.

The so-called modified diffraction intensity is defined as


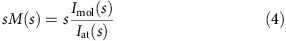


The spatial resolution, *δ*, is determined by the maximum measured *s* value *s*_max_ using the formula


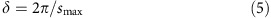


**Figure 2:**
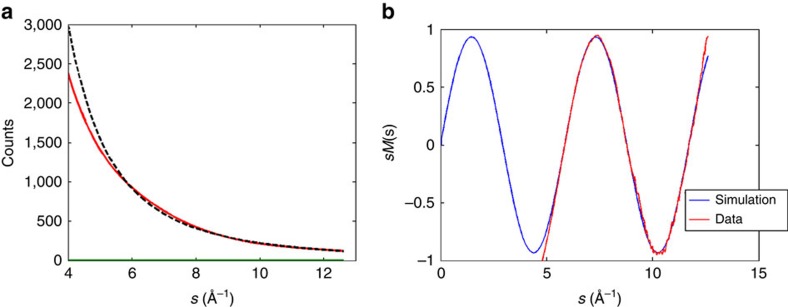
Static N_2_ diffraction. (**a**) The red curve shows an azimuthally averaged raw experimental diffraction pattern after subtraction with a dark background pattern that is taken without the electron beam. The green curve shows the azimuthal average of the subtracted dark background pattern. The dashed black curve shows the fitted background, including the atomic scattering *I*_at_ and other background scattering. The vertical axis is average detector counts per pixel per minute of exposure time, averaged over ∼100 min. The green curve varies between 0.8 and 2.5 counts. (**b**) The theoretical (blue) and experimental (red) modified diffraction intensity *sM* from N_2_ gas, which shows the enhanced diffraction rings. The experimental *sM* is calculated from the diffraction pattern in part (**a**).

In Fig. 2a the red curve shows an azimuthally averaged raw pattern after subtraction with a dark background pattern that is taken with the electron beam turned off. The azimuthal average of the subtracted background pattern is shown in the green curve in Fig. 2a, and the black dashed curve shows the experimental scattering background, determined using a standard fitting procedure^20^. This fitted background includes the atomic scattering *I*_at_, scattering from background gas and other types of background scatterings. The details of the bond length determination are explained in the Bond Length Determination from Static Pattern section in the Methods. Figure 2b shows the azimuthally averaged experimental and theoretical modified diffraction intensity for static diffraction pattern. The bond length was determined to be 1.073±0.027 Å, in agreement with the previously measured N_2_ bond length of 1.098 Å (ref. 58). The 2.5% measurement uncertainty is due to the uncertainty in the calibration of the sample-to-detector distance and electron energy, as explained in the Experimental Setup section in the Methods. It should be noted that for time-resolved experiments usually the ground-state structure is known, in which case the static diffraction patterns can be used as a calibration. We show later that the interatomic distance can be determined very accurately from diffraction of transiently aligned molecules if the static diffraction is used as a calibration. In this experiment, scattering signal is available in the region between 3.5 and 12 Å^−1^. The fitting procedure relies on the zeros of *sM*(*s*) and this reduces the available data in Fig. 2 to *s*>4.5 Å^−1^. The measurement agrees well with the simulation up to *s*∼12 Å^−1^.

### Temporal evolution of N_2_ alignment

For impulsive alignment, the full rotational revival is expected at *t*=1/2*cB*, where *B* is the rotational constant and *c* is the speed of light in vacuum. For N_2_ molecules, the full revival is at 8.35 ps (*B*=1.998 cm^−1^). The rapid evolution of the angular distribution can be used to determine the temporal resolution of the measurement technique.

**Figure 3:**
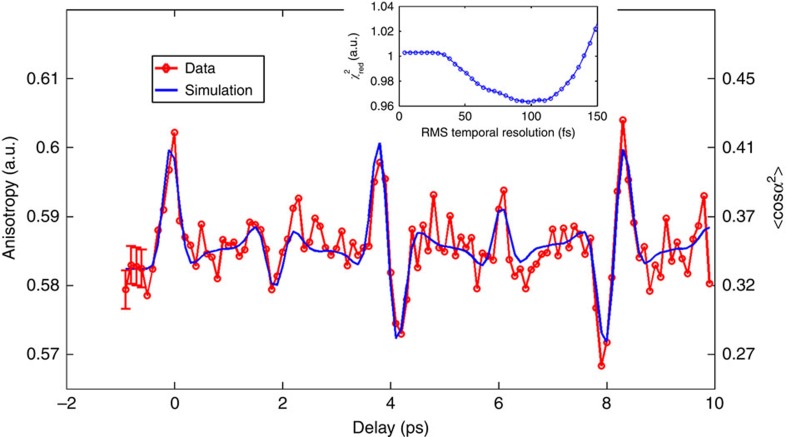
Temporal evolution of the N_2_ rotational wavepacket. Anisotropy in the diffraction patterns from experimental data (red) and simulation (blue) versus time. Statistical error bars for the first few points (before alignment) are shown to illustrate the uncertainty of this measurement. The right-hand side axis gives the degree of alignment <cos^2^*α*> for the simulated curve. The simulation parameters of initial rotational temperature, alignment laser fluence, temporal resolution and rescaling factor are obtained from a fitting routine. Each data point is accumulated over 2 min. Reduced *χ*^2^ error versus RMS temporal resolution in the four-parameter fit is shown in the inset.

Diffraction patterns from a molecular ensemble aligned with a polarization in the detector plane are not circularly symmetric, contrary to the static case in equation (3). The anisotropy, *a*(*t*), in a diffraction pattern can be used to trace the temporal evolution of alignment^49^. In addition, *a*(*t*) is a self-normalized parameter that is extracted directly from the diffraction patterns (see Data Processing of Diffraction Patterns and Anisotropy in the Methods). Figure 3 shows the temporal evolution of the simulated and experimentally measured *a*(*t*) values. The experimental data are recorded with 100 fs steps in the delay between laser and electron pulses, and at each point data are collected for 2 min (14,400 shots at a repetition rate of 120 Hz). The simulation is composed of two parts: an impulsive alignment simulation that calculates the angular distribution at different delay times^59^, followed by a diffraction pattern simulation based on the modelled angular distribution^60^. The anisotropy of the simulated patterns is calculated using the same method as for the experimental patterns. The details of the simulation are explained in the Alignment Simulation and Diffraction Pattern Simulation section in the Methods. For diatomic molecules, the rotational states can be described by |*J*, *M*>, where *J* and *M* are the quantum numbers of the total and the z-component of the angular momentum, respectively. The angular distribution of |*J*, *M*> is given by Laplace's spherical harmonics 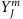. Before the excitation, the ensemble is described by a Boltzmann distribution of rotational states taking into account the exact nuclear statistics. Initially, the angular distribution is isotropic. For the initial rotational temperature of our sample of 54 K, all values of *J* up to 10 are considerably populated before excitation. The laser pulse interacts with the polarizability tensor of the molecules, which is highest along the internuclear axis. Classically, it induces a dipole that minimizes its energy by aligning the molecular axis parallel to the laser field. Quantum mechanically, the laser induces transitions of Δ*J*=0, ±2 and Δ*M*=0. After excitation, the maximum value of *J* is about 14. The phase of each rotational state evolves with time. When the rotational states are in phase, the angular distribution becomes aligned or anti-aligned with respect to the laser polarization axis.

The temporal evolution of the alignment has a rich structure that varies on a fast timescale. For example, at the half-revival (delay of 4 ps), the distribution changes from aligned to anti-aligned in 300 fs. The alignment peak corresponds to a prolate angular distribution, with the long axis along the direction of the laser polarization. The angular distribution during anti-alignment is oblate, with the molecules preferentially lying in a plane perpendicular to the laser polarization. The full revival at around 8 ps shows a similarly fast transition from oblate to prolate distribution. In between the revivals, the anisotropy of the diffraction pattern captures additional dynamics.

We have used the fast-changing distribution to characterize the temporal resolution of the measurement. The shape of the aligned molecular ensemble is determined by the initial temperature of the molecules and the fluence of the alignment laser pulse^61^. In this experiment, the laser fluence was measured to be 2.0 J cm^−2^ and the initial temperature was estimated to be 65 K, using a supersonic expansion model^62^. At these parameters, the wavepacket revivals are relatively sharp compared with the temporal resolution. The limited temporal resolution effectively blurs out the rotational dynamics and has a significant effect on the observed structure. We performed first a two-parameter *χ*^2^ fitting by fixing the laser fluence and initial rotational temperature to the measured and calculated values, and then a full four-parameter fit where all parameters were allowed to vary.

The two-parameter fit, varying only the temporal resolution and a re-scaling factor that accounts for the spatial overlap between laser and electron pulse, returned a temporal resolution of 85 fs RMS (200 fs FWHM). The four-parameter fit, which varies temporal resolution in addition to initial rotational temperature, laser fluence and the re-scaling factor, accounts for uncertainties in the laser fluence, initial temperature and the fraction of molecules excited by the laser. In the simulation, we assume that all excited molecules are exposed to the same laser intensity. This method achieved a best fit resolution of 100 fs RMS (230 fs FWHM), with an initial temperature of 54 K and a laser fluence of 1.8 J cm^−2^, which are comparable to our initial estimates for the two-parameter fit. The re-scaling factor was 0.42, meaning that the best match to the experimental data is when the simulation assumes that 42% of the molecules in the diffraction volume are excited by the laser. The simulation shown in Fig. 3 shows the best fit for all four parameters. The left axis in the figure shows the anisotropy in the diffraction pattern, and the axis on the right shows the degree of alignment quantified by the <cos^2^*α*> value from simulation, where *α* is the angle between the molecular axis and the laser polarization direction. The reduced *χ*^2^ error in the four-parameter fit versus the RMS temporal resolution is shown as an inset in Fig. 3. We obtained a 0.96 reduced *χ*^2^, indicating a good fit. More details of the fitting are explained in the Temporal Evolution Fitting section in the Methods.

The 100-fs (RMS) overall temporal resolution of this experiment is consistent with our expectations, based on the performance study of this machine^56^. A simulation showed that the electron bunch length was 70 fs RMS at the interaction region. Measurements of the phase and amplitude stability of the radio-frequency (RF) gun lead to an expected time of arrival jitter of 50 fs RMS^56^. The calculated overall temporal resolution was then 87 fs RMS, or 205 fs FWHM, which is close to the measured value.

### Molecular images with different angular distribution

High-resolution molecular diffraction images were retrieved for prolate and oblate ensembles at the half revival. Diffraction patterns with adequate signal-to-noise ratio were recorded with 60 and 90 min of integration time for oblate and prolate distributions, respectively. We use diffraction-difference patterns to remove the experimental background and the diffraction signal from unexcited molecules. The diffraction intensity difference is given by Δ*I*(*t*)=*I*(*t*)−*I*(*t*=−5 ps), where *t*=0 corresponds to the maximum of the first alignment peak after laser. Before *t*=−0.4 ps when the pump laser arrives, the angular distribution is isotropic. In the two-dimensional (2D) diffraction patterns, the *s*_max_=8.3 Å^−1^, corresponding to a spatial resolution of 0.76 Å. This makes it possible to observe molecular structures with resolution better than the shortest possible bond lengths.

**Figure 4:**
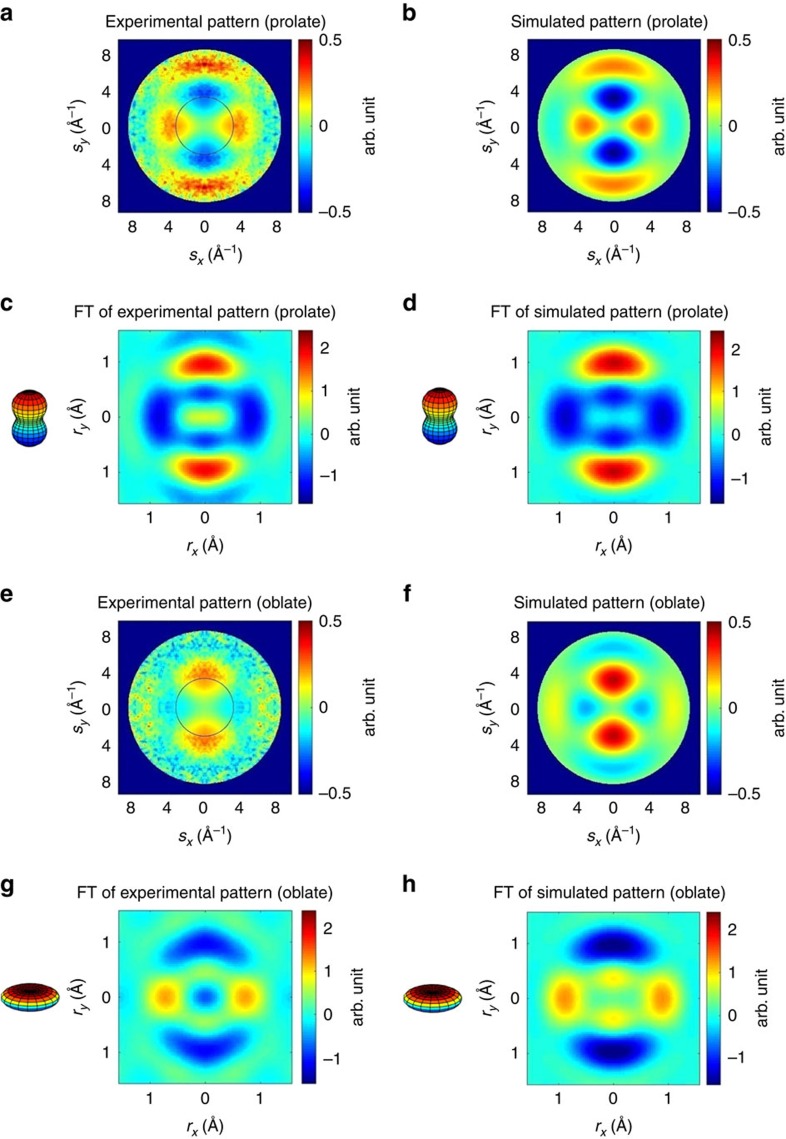
2D N_2_ diffraction patterns at half revival. (**a**) Experimentally measured and (**b**) simulated diffraction-difference patterns of the prolate distribution. Images shown in **c**,**d** are Fourier transforms of **a**,**b**, respectively. The Fourier transform of the diffraction-difference patterns show the changes in the angular distribution of the molecules. The positive regions (red colour) indicate where the population has increased and the negative regions (blue colour) indicate where the population has decreased. (**e**) Experimentally measured and (**f**) simulated diffraction-difference pattern of the oblate distribution. Images shown in **g**,**h** are Fourier transforms of **e**,**f**, respectively. In patterns (**a**,**e**), the data inside the black circles are missing due to the beam stop. They are obtained by extrapolating the pattern and letting the counts smoothly go to zero towards the centre. For illustrative purpose, angular distributions are shown on the side of panel (**c**,**d**,**g**,**h**) for visual guidance. In these angular distributions, the colour code indicates polar angle.

Figure 4 shows the experimental (left panels) and simulated (right panels) 2D diffraction patterns and their corresponding Fourier transforms for prolate and oblate distribution at the half revival. Figure 4a,b shows the experimental and simulated diffraction-difference patterns Δ*I* for the prolate distribution. The diffraction pattern was captured at a time delay of 3.8 ps after the first alignment peak (Fig. 3a). The diffraction-difference pattern is anisotropic as a result of molecular alignment. The experimental pattern shows excellent agreement with the simulation for a range of *s*=3.5–8.3 Å^−1^.

Figure 4c,d shows the Fourier transforms of the difference signals in Fig. 4a,b, respectively. The Fourier transform of the diffraction pattern displays the autocorrelation of the molecular structure, convolved with the angular distribution and projected onto the detector plane. The centre of the Fourier transforms goes to zero because they are generated from the difference of two diffraction patterns. For a diatomic molecule, the autocorrelation is directly related to the molecular image. For more complex molecules, an image of the structure can be reconstructed using phase retrieval algorithms^60^. In the autocorrelation functions depicted in Fig. 4c,d, the positive regions indicate an increase in population, and the negative regions indicate a decrease. Specifically, Fig. 4c,d indicates that the population of molecules that are lying perpendicular to the laser polarization has decreased, whereas the population parallel to the polarization (vertical in Fig. 4) has increased, that is, more molecules are aligned along the vertical direction. Similarly, Fig. 4e,f shows the measured and simulated diffraction pattern for the oblate distribution at half revival, corresponding to a time delay of 4.1 ps. Figure 4g,h are the Fourier transforms of Fig. 4e,f, which show that the molecular ensemble is aligned in the horizontal plane.

### Bond length measurement from diffraction patterns

We have used a fitting method to extract the N_2_ bond length from diffraction patterns of aligned and anti-aligned molecules, using the static diffraction pattern as a calibration. The details of the fitting are given in the section Bond Length Fitting from Aligned Patterns in the Methods. The extracted bond length is 1.091±0.036 Å for the prolate distribution and 1.096±0.056 Å for the oblate distribution, in good agreement with 1.098 Å, the bond length of the ground-state N_2_. The small uncertainties indicate that changes in interatomic distances could be measured very accurately with this method. The precision of determining the bond length, 0.036 and 0.056 Å, should not be confused with the 0.76 Å spatial resolution. The spatial resolution gives the capability to distinguish two bond lengths that are very close to each other, whereas the precision gives how accurate a single bond length can be determined. In real space, the resolution is determined by the width of the peak, whereas the precision is determined by how accurate one can find the centre of the peak. Generally, for well-separated peaks, the centre can be determined to much higher accuracy than its width.

### Spatial resolution of the molecular images

The spatial resolution of the 2D diffraction patterns shown in Fig. 4 can be determined in two different ways. First we can use equation (6), with *s*_max_=8.3 Å^−1^ we get the spatial resolution *δ*=0.76 Å. We can also determine spatial resolution directly from the autocorrelation images (Fig. 4c,g). For example, in Fig. 4c, the spatial resolution can be determined by converting the image into polar coordinates *o*(*r, θ'*), then using a Gaussian function to fit to along the *r* dimension. The FWHM of the Gaussian fit was 0.76 Å using this method, consistent with the spatial resolution obtained using equation (5) and *s*_max_=8.3 Å^−1^.

### Angular distribution based on 2D images of aligned molecules

**Figure 5:**
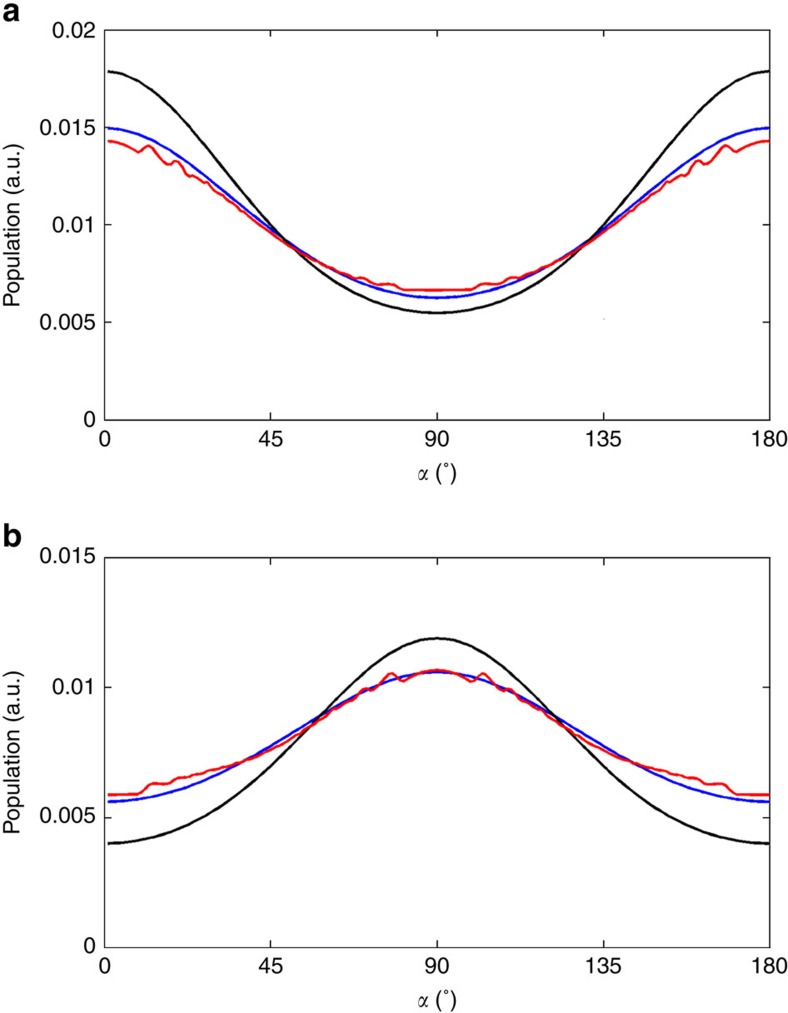
Angular distributions. (**a**) Prolate angular distribution at the revival: experimental (red), simulated (black) and simulated convolved with 100 fs RMS temporal resolution (blue). The effect of the temporal resolution is to reduce the measured degree of alignment at the peak, due to time averaging. The measurement agrees very well with the simulation after the convolution. The <cos^2^*α*> values for red, black and blue curves are 0.41, 0.45 and 0.42, respectively. (**b**) Oblate angular distribution: Experimental (red), simulated (black) and simulated convolved with 100 fs RMS temporal resolution (blue). The <cos^2^*α*> values for red, black and blue curves are 0.28, 0.25 and 0.28, respectively. The experimental curves are measured from Fig. 4c,g, respectively.

The angular distribution of the molecules can be extracted from the patterns in Fig. 4c,g. The resulting distributions for prolate and oblate molecular ensembles are shown in Fig. 5a,b, respectively (see Angular Distribution section in the Methods). The prolate distribution peaks at *α*=0 and 180°, in the direction of the laser polarization. The oblate distribution peaks at *α*=90°, in the direction perpendicular to the laser polarization. The degree of alignment is commonly measured by the quantity


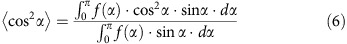


where *f*(*α*) is the angular distribution and *α* is the angle between the molecular axis and the laser polarization. A value of the alignment parameter <cos^2^*α*>=1 corresponds to perfect alignment, whereas random orientation gives <cos^2^*α*>=1/3. Any value between 1/3 and 1 indicates alignment, whereas any value below 1/3 indicates anti-alignment. In Fig. 5, three curves are displayed: *f*(*α*) extracted from data (red), *f*(*α*) obtained from simulation using the fluence and temperature extracted from the fitting routine (black), and the simulation results convolved with the 100 fs RMS temporal resolution (blue). The temporal resolution is included by convolving a Gaussian pulse with the simulated temporal evolution of the angular distribution, and it has a significant effect because the distribution changes from prolate to oblate in 300 fs. The extracted angular distributions are in good agreement with the simulation for both prolate and oblate distributions, after taking into account the effect of the temporal resolution.

The measured angular distribution can also be used to provide additional confirmation of the temporal resolution. Using the laser parameters and the initial temperature as determined by the fitting from the data in Fig. 3, we ran an optimization to find the temporal convolution that results in a best fit between the measured and simulated angular distributions. From this, we obtain a resolution of 107 fs RMS for the prolate angular distribution and 102 fs RMS for the oblate distribution, in good agreement with the value of 100 fs RMS obtained before.

## Discussion

In summary, we have shown that MeV UED can simultaneously reach a temporal resolution of 100 fs RMS and spatial resolution of 0.76 Å, which allows us to characterize the ultrafast evolution of a rotational wavepacket in N_2_, measuring the angular distribution and the acquired molecular images with atomic resolution.

This achievement opens the door to a new class of experiments where changes in molecular geometry during a chemical reaction can be followed in space and time. The current results demonstrate not only excellent temporal resolution, but also that sufficient signal-to-noise ratio for sub-Angstrom spatial resolution can be acquired with MeV electrons. Previous picosecond UED experiments with similar spatial resolution have successfully retrieved the structure of transient states^15^. Nitrogen has a low atomic number, *Z*, and therefore a low scattering cross-section, so we expect that the method will be successful for observing molecular dynamics in a large class of molecules. RF-compressed electron pulses^63^,^64^ and compact UED guns^65^,^66^,^67^ have achieved similar temporal resolution in condensed matter experiments, but have not yet been successfully applied to gas-phase experiments. For future developments, a number of upgrades can potentially improve both spatial and temporal resolution significantly. For example, a RF compression cavity could be used to compress the electron beam longitudinally^68^, which can potentially lead to a temporal resolution on the order of 10 fs together with orders-of-magnitude increase in charge per pulse. Time jitter and drift could be addressed by time-stamping techniques similar to those developed for X-Ray free electron lasers (XFELs)^69^. Spatial resolution is limited by the signal-to-noise ratio (SNR) in the scattering at larger angles, so RF compression would also improve the spatial resolution by increasing the number of electrons per pulse. Complementary metal-oxide semiconductor (CMOS) active pixel sensor could potentially achieve single electron, single shot detection and thus eliminate detector noise^70^, which would also improve the SNR.

## Methods

### Experimental setup

The electron gun used in this experiment is a replica of the photo-injector used at the LCLS facility at SLAC National Accelerator Laboratory. A 15 fC per pulse charge is generated at the photocathode and 6 fC per pulse is delivered on target. A 50-nm-thick Si_3_N_4_ membrane is used to separate the sample chamber and preserve the high vacuum in the electron gun. Roughly 60% of the charge is lost after the Si_3_N_4_ membrane and a 200-μm diameter collimator. The electron beam and pump laser are generated with a repetition rate of 120 Hz.

The N_2_ gas is delivered into the chamber through a pulsed valve at a 120-Hz repetition rate. The backing pressure is 0.7 bar, and the nozzle orifice is 100 μm in diameter. During operation, the chamber pressure is ∼6 × 10^−5^ torr. The interaction region is roughly 350 μm away from the nozzle exit. The width of the laser beam, the electron beam and the gas jet are all around 200 μm FWHM at the interaction region. The sample-to-detector distance is calibrated with diffraction from a single crystal gold sample. There is a ±2.5% uncertainty in the distance calibration, due to the quality of the gold sample and quality of the electron beam after the Si_3_N_4_ membrane. The Si_3_N_4_ window has a strong diffraction and makes the emittance of the beam much worse. The electrons scattered inelastically from Si_3_N_4_ also diffract from the gold sample, making the Bragg spots larger. Spatial alignment of the gas jet, the electron beam and the pump laser is obtained by positioning the focus of the laser approximately underneath the tip of the nozzle, and maximizing for the electron beam deflection by the plasma. Time zero can then be found, to within ∼200 fs, by adjusting the delay between the pump and the probe beams and observing plasma deflection effects on the unscattered beam^20^. The laser focus is adjusted to 50 μm FWHM spot size for plasma lensing, giving a peak intensity of 8 × 10^14^ W cm^−2^. For the alignment experiments, the lens is driven 4 mm out of the focus to reach a spot size of 200 μm in the interaction region. The peak intensity for the experiment is 5 × 10^13^ W cm^−2^; only very faint plasma is observed in the interaction region for this intensity.

The detector comprises a phosphor screen, a ring-shaped mirror at a 45° angle to the beam, an f/0.85 lens and an electron multiply charge-coupled device. A 4-mm diameter hole is drilled in the centre of the phosphor screen to allow the unscattered electron beam to pass through. Data in the region *s*<3.5 Å^−1^ are not captured due to the hole in the phosphor screen. This can be improved by replacing the phosphor screen by a similar one with a smaller hole. We have seen that by replacing the phosphor screen the missing region can be reduced to 1.6 Å^−1^.

The beam divergence *σ* is 28 μrad RMS. Using the definition *λ*/2*πσ*, we get a transverse coherence length of 1.7 nm.

### Bond length determination from static pattern

The following steps are used to determine the bond length from the static N_2_ diffraction pattern^20^. (i)Starting from an initial guess of the bond length the modified diffraction intensity *sM* is calculated. (ii) A series of zero points is determined from the simulated *sM*. (iii) The background is determined by fitting an exponentially decaying function at the zero points. (iv) The experimental *sM* is obtained by subtracting the background. (v) The experimental *sM* is compared with the simulated *sM* and the error is defined as the square of the difference. (vi) Steps i–iv are repeated for different bond lengths, until the minimum error is found.

### Data processing of diffraction patterns and anisotropy

The experimental pattern is symmetrized over four quadrants, and the central region (*s*<3.5 Å^−1^) is extrapolated from existing data by letting the pattern smoothly go to zero towards the centre.

The diffraction pattern of the prolate distribution is integrated over 90 min, and that of the oblate distribution is integrated over 60 min. The anisotropy is calculated by dividing the total counts in a horizontal cone by the total counts in a vertical cone for each diffraction pattern. The region used for this calculation is between *s*=3 Å^−1^ and *s*=4.5 Å^−1^. The horizontal cone has a half angle of 35° and the vertical cone has a half angle of 55°.

### Alignment simulation

The simulation of impulsive alignment is calculated using a linear rigid rotor interacting with a non-resonant pulse described by the time-dependent Schrodinger equation^59^. The temporal resolution is implemented by a convolution with a Gaussian function in time.

### Diffraction pattern simulation

The simulations of diffraction patterns of a given angular distribution are calculated using an incoherent weighted sum of diffraction patterns from single molecules with different orientations. In the simulations, the atoms are assumed to be stationary at their equilibrium positions without vibrations^60^.

### Temporal evolution fitting

The temporal evolution fitting shown in Fig. 3a is obtained by a *χ*^2^ fitting of the simulation and data. The simulated anisotropy is obtained by an alignment simulation that gives angular distribution as a function of time, followed by a diffraction simulation according to the angular distribution. The anisotropy is calculated using the same method as for data, and the temporal resolution is obtained by a convolution of the calculated anisotropy with a Gaussian beam in time. The reduced *χ*^2^ error, plotted in the Fig. 3 inset, is defined as 
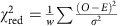
, where *w* is the number of degrees of freedom, *O* and *E* are the data and the best fit, *σ* is the s.e., respectively. Here *σ* is calculated using the standard deviation of 13 patterns before *t*=0.

### Bond length fitting from aligned patterns

The optimal bond length is found by comparing the experimental diffraction pattern with many simulated patterns with different bond lengths using the least-square error. For the experimental patterns, a vertical cone with a half opening angle of 30° is selected, and this part is averaged radially to generate a one-dimensional curve. For the simulated patterns, diffraction patterns with different bond lengths and the same angular distribution are simulated, and the same region is selected to generate the one-dimensional curve. The prolate/oblate angular distributions are obtained by the fitting in Fig. 3. To get statistics, ten independent data set (6 min data in each set) were used in the fits separately, and the mean and standard deviation of the ten fitting results are taken as the final result and the standard error. In this fitting, we use the static pattern (shown in Fig. 2) as a calibration of the sample-detector distance and electron beam energy.

### Angular distribution

The angular distribution in Fig. 5a,b are extracted from Fig. 4c,g, respectively. The procedure includes three steps: (i) extracting the difference angular distribution from the 2D difference pattern, (ii) adding a baseline that accounts for the angular distribution of the reference pattern to the difference in angular distribution and (iii) normalizing the angular distribution. Step i is implemented by first converting Fig. 4c,g to polar coordinates, then integrating over the radial coordinate within the FWHM of the peak. In step ii, the baseline is simulated using equations (2) and (3), corrected by the rescaling parameter that accounts for the spatial overlap between laser and electrons (0.42, obtained from the fitting for Fig. 3). In step iii, the normalization used is 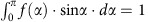.

## Additional information

**How to cite this article:** Yang, J. *et al.* Diffractive imaging of a rotational wavepacket in nitrogen molecules with femtosecond megaelectronvolt electron pulses. *Nat. Commun.* 7:11232 doi: 10.1038/ncomms11232 (2016).

## References

[b1] Pedersen S., Herek J. L. & Zewail A. H. The validity of the ‘diradical' hypothesis: direct femtoscond studies of the transition-state structures. Science 266, 1359–1364 (1994) . 17772843 10.1126/science.266.5189.1359

[b2] Schoenlein R. W., Peteanu L. A., Mathies R. A. & Shank C. V. The first step in vision: femtosecond isomerization of rhodopsin. Science 254, 412–415 (1991) . 1925597 10.1126/science.1925597

[b3] Mathies R. A., Brito Cruz C. H., Pollard W. T. & Shank C. V. Direct observation of the femtosecond excited-state cis-trans isomerization in bacteriorhodopsin. Science 240, 777–779 (1988) . 3363359 10.1126/science.3363359

[b4] Blanchet V., Zgierski M. Z., Seideman T. & Stolow A. Discerning vibronic molecular dynamics using time-resolved photoelectron spectroscopy. Nature 401, 52–54 (1999) .

[b5] Bisgaard C. Z. *et al.* Time-resolved molecular frame dynamics of fixed-in-space CS_2_ molecules. Science 323, 1464–1468 (2009) . 19286552 10.1126/science.1169183

[b6] Deb S. & Weber P. M. The ultrafast pathway of photon-induced electrocyclic ring-opening reactions: the case of 1,3-cyclohexadiene. Annu. Rev. Phys. Chem. 62, 19–39 (2011) . 21054174 10.1146/annurev.physchem.012809.103350

[b7] Gessner O. *et al.* Femtosecond multidimensional imaging of a molecular dissociation. Science 311, 219–222 (2006) . 16357226 10.1126/science.1120779

[b8] Milne C. J., Penfold T. J. & Chergui M. Recent experimental and theoretical developments in time-resolved X-ray spectroscopies. Coord. Chem. Rev. 277, 44–68 (2014) .

[b9] Küpper J. *et al.* X-ray diffraction from isolated and strongly aligned gas-phase molecules with a free-electron laser. Phys. Rev. Lett. 112, 83002 (2014) .

[b10] Ihee H. *et al.* Ultrafast x-ray diffraction of transient molecular structures in solution. Science 309, 1223–1227 (2005) . 16020695 10.1126/science.1114782

[b11] Siders C. W. *et al.* Detection of nonthermal melting by ultrafast X-ray diffraction. Science 286, 1340–1342 (1999) . 10558985 10.1126/science.286.5443.1340

[b12] Minitti M. P. *et al.* Imaging molecular motion: femtosecond X-ray scattering of an electrocyclic chemical reaction. Phys. Rev. Lett. 114, 255501 (2015) . 26197134 10.1103/PhysRevLett.114.255501

[b13] Gao M. *et al.* Mapping molecular motions leading to charge delocalization with ultrabright electrons. Nature 496, 343–346 (2013) . 23598343 10.1038/nature12044

[b14] Siwick B. J., Dwyer J. R., Jordan R. E. & Miller R. J. D. An atomic-level view of melting using femtosecond electron diffraction. Science 302, 1382–1385 (2003) . 14631036 10.1126/science.1090052

[b15] Ihee H. *et al.* Direct imaging of transient molecular structures with ultrafast diffraction. Science 291, 458–462 (2001) . 11161194 10.1126/science.291.5503.458

[b16] Srinivasan R., Feenstra J. S., Park S. T., Xu S. & Zewail A. H. Dark structures in molecular radiationless transitions determined by ultrafast diffraction. Science 307, 558–563 (2005) . 15637234 10.1126/science.1107291

[b17] Blaga C. I. *et al.* Imaging ultrafast molecular dynamics with laser-induced electron diffraction. Nature 483, 194–197 (2012) . 22398558 10.1038/nature10820

[b18] Meckel M. *et al.* Laser-induced electron tunneling and diffraction. Science 320, 1478–1482 (2008) . 18556555 10.1126/science.1157980

[b19] Krasniqi F. *et al.* Imaging molecules from within: ultrafast angström-scale structure determination of molecules via photoelectron holography using free-electron lasers. Phys. Rev. A At. Mol. Opt. Phys. 81, 1–11 (2010) .

[b20] Srinivasan R., Lobastov V. A., Ruan C.-Y. & Zewail A. H. Ultrafast electron diffraction (UED): a new development for the 4D determination of transient molecular structures. Helv. Chim. Acta 86, 1763–1838 (2003) .

[b21] Yang J., Beck J., Uiterwaal C. J. & Centurion M. Imaging of alignment and structural changes of carbon disulfide molecules using ultrafast electron diffraction. Nat. Commun. 6, 8172 (2015) . 26337631 10.1038/ncomms9172

[b22] Sciaini G. & Miller R. J. D. Femtosecond electron diffraction: heralding the era of atomically resolved dynamics. Rep. Prog. Phys. 74, 096101 (2011) .

[b23] Miller R. J. D. Mapping atomic motions with ultrabright electrons: The Chemists' Gedanken Experiment Enters the Lab Frame. Annu. Rev. Phys. Chem. 65, 583–604 (2014) . 24423377 10.1146/annurev-physchem-040412-110117

[b24] Levine B. G. & Martínez T. J. Isomerization through conical intersections. Annu. Rev. Phys. Chem. 58, 613–634 (2007) . 17291184 10.1146/annurev.physchem.57.032905.104612

[b25] Kochman M. A., Tajti A., Morrison C. A. & Miller R. J. D. Early events in the nonadiabatic relaxation dynamics of 4-(N, N -dimethylamino)benzonitrile. J. Chem. Theory Comput. 11, 1118–1128 (2015) . 26579762 10.1021/ct5010609

[b26] Ischenko A. A., Ewbank J. D. & Lothar S. Structural kinetics by stroboscopic gas electron diffraction Part 1. Time-dependent molecular intensities of dissociative states. J. Mol. Struct. 320, 147–158 (1994) .

[b27] Elsayed-Ali H. E. & Mourou G. a. Picosecond reflection high-energy electron diffraction. Appl. Phys. Lett. 52, 103–104 (1988) .

[b28] Ruan C. Y. *et al.* Ultrafast diffraction and structural dynamics: the nature of complex molecules far from equilibrium. Proc. Natl Acad. Sci. USA 98, 7117–7122 (2001) . 11404473 10.1073/pnas.131192898 PMC34632

[b29] Schultz T. *et al.* Mechanism and dynamics of azobenzene photoisomerization. J. Am. Chem. Soc. 125, 8098–8099 (2003) . 12837068 10.1021/ja021363x

[b30] Crespo-Hernandez C., Cohen B., Hare P. & Kohler B. Ultrafast excited-state dynamics in nucleic acids. Chem. Rev. 104, 1977–2019 (2004) . 15080719 10.1021/cr0206770

[b31] McFarland B. K. *et al.* Ultrafast X-ray Auger probing of photoexcited molecular dynamics. Nat. Commun. 5, 4235 (2014) . 24953740 10.1038/ncomms5235

[b32] Middleton C. T. *et al.* DNA excited-state dynamics: from single bases to the double helix. Annu. Rev. Phys. Chem. 60, 217–239 (2009) . 19012538 10.1146/annurev.physchem.59.032607.093719

[b33] Schreier W. J. *et al.* Thymine dimerization in DNA is an ultrafast photoreaction. Science 315, 625–629 (2007) . 17272716 10.1126/science.1135428 PMC2792699

[b34] Siwick B. J., Dwyer J. R., Jordan R. E. & Miller R. J. D. Ultrafast electron optics: propagation dynamics of femtosecond electron packets. J. Appl. Phys. 92, 1643–1648 (2002) .

[b35] Dantus M., Kim S. B., Williamson J. C. & Zewail A. H. Ultrafast electron diffraction. 5. Experimental time resolution and applications. J. Phys. Chem. 98, 2782–2796 (1994) .

[b36] Hastings J. B. *et al.* Ultrafast time-resolved electron diffraction with megavolt electron beams. Appl. Phys. Lett. 89, 184109 (2006) .

[b37] Li R. *et al.* Experimental demonstration of high quality MeV ultrafast electron diffraction. Rev. Sci. Instrum 80, 083303 (2009) . 19725647 10.1063/1.3194047

[b38] Zhu P. *et al.* Femtosecond time-resolved MeV electron diffraction. N. J. Phys. 17, 063004 (2015) .

[b39] Wang X., Qiu X. & Ben-Zvi I. Experimental observation of high-brightness microbunching in a photocathode rf electron gun. Phys. Rev. E 54, R3121–R3124 (1996) . 10.1103/physreve.54.r3121 9965628

[b40] Wang X. J., Wu Z. & Ihee H. Femto-seconds electron beam diffraction using photocathode RF gun. Proc. 2003 Part Accel. Conf. 1, 420–422 (2003) .

[b41] Wang X. J., Xiang D., Kim T. K. & Ihee H. Potential of femtosecond electron diffraction using near-relativistic electrons from a photocathode RF electron gun. J. Korean Phys. Soc. 48, 390–396 (2006) .

[b42] Musumeci P., Moody J. T. & Scoby C. M. Relativistic electron diffraction at the UCLA Pegasus photoinjector laboratory. Ultramicroscopy 108, 1450–1453 (2008) . 18640780 10.1016/j.ultramic.2008.03.011

[b43] Muro'Oka Y. *et al.* Transmission-electron diffraction by MeV electron pulses. Appl. Phys. Lett. 98, 2009–2012 (2011) .

[b44] Fu F. *et al.* High quality single shot ultrafast MeV electron diffraction from a photocathode radio-frequency gun. Rev. Sci. Instrum 85, 083701 (2014) . 25173270 10.1063/1.4892135

[b45] Manz S. *et al.* Mapping atomic motions with ultrabright electrons: towards fundamental limits in space-time resolution. Faraday Discuss. 177, 467–491 (2015) . 25631530 10.1039/c4fd00204k

[b46] Reiser M. Theory and Design of Charged Particle Beams Wiley (1994) .

[b47] Stapelfeldt H. & Seideman T. Colloquium: aligning molecules with strong laser pulses. Rev. Mod. Phys. 75, 543–557 (2003) .

[b48] Rosca-Pruna F. & Vrakking M. J. Experimental observation of revival structures in picosecond laser-induced alignment of I_2_. Phys. Rev. Lett. 87, 153902 (2001) . 11580701 10.1103/PhysRevLett.87.153902

[b49] Hensley C. J., Yang J. & Centurion M. Imaging of isolated molecules with ultrafast electron pulses. Phys. Rev. Lett. 109, 133202 (2012) . 23030087 10.1103/PhysRevLett.109.133202

[b50] Reckenthaeler P. *et al.* Time-resolved electron diffraction from selectively aligned molecules. Phys. Rev. Lett. 102, 213001 (2009) . 19519100 10.1103/PhysRevLett.102.213001

[b51] Chen Y.-H., Varma S. & Milchberg H. M. Space- and time-resolved measurement of rotational wave packet revivals of linear gas molecules using single-shot supercontinuum spectral interferometry. J. Opt. Soc. Am. B 25, B122 (2008) .

[b52] Litvinyuk I. V. *et al.* Alignment-dependent strong field ionization of molecules. Phys. Rev. Lett. 90, 233003 (2003) . 12857255 10.1103/PhysRevLett.90.233003

[b53] Itatani J. *et al.* Controlling high harmonic generation with molecular wave packets. Phys. Rev. Lett. 94, 123902 (2005) . 15903921 10.1103/PhysRevLett.94.123902

[b54] McFarland B. K., Farrell J. P., Bucksbaum P. H. & Gühr M. High harmonic generation from multiple orbitals in N_2_. Science 322, 1232–1235 (2008) . 18974318 10.1126/science.1162780

[b55] Cryan J. P. *et al.* Auger electron angular distribution of double core-hole states in the molecular reference frame. Phys. Rev. Lett. 105, 083004 (2010) . 20868096 10.1103/PhysRevLett.105.083004

[b56] Weathersby S. P. *et al.* Mega-electron-volt ultrafast electron diffraction at SLAC National Accelerator Laboratory. Rev. Sci. Instrum. 86, 073702 (2015) . 26233391 10.1063/1.4926994

[b57] Salvat F., Jablonski A. & Powell C. J. Elsepa—Dirac partial-wave calculation of elastic scattering of electrons and positrons by atoms, positive ions and molecules. Comput. Phys. Commun. 165, 157–190 (2005) .

[b58] Huber K. P. & Herzberg G. in Molecular Spectra and Molecular Structure IV. Constants of Diatomic Molecules Springer (1979) .

[b59] Ortigoso J., Rodriguez M., Gupta M. & Friedrich B. Time evolution of pendular states created by the interaction of molecular polarizability with a pulsed nonresonant laser field. J. Chem. Phys. 110, 3870–3875 (1999) .

[b60] Yang J., Makhija V., Kumarappan V. & Centurion M. Reconstruction of three-dimensional molecular structure from diffraction of laser-aligned molecules. Struct. Dyn. 1, 044101 (2014) . 26798781 10.1063/1.4889840 PMC4711636

[b61] Holmegaard L. *et al.* Control of rotational wave-packet dynamics in asymmetric top molecules. Phys. Rev. A 75, 051403 (2007) .

[b62] Hagena O. F. Nucleation and growth of clusters in expanding nozzle flows. Surface Sci. Lett. 106, 101–116 (1981) .

[b63] Chatelain R. P., Morrison V. R., Godbout C. & Siwick B. J. Ultrafast electron diffraction with radio-frequency compressed electron pulses. Appl. Phys. Lett. 101, 2–6 (2012) . 10.1103/PhysRevLett.113.235502 25526134

[b64] Van Oudheusden T. *et al.* Compression of subrelativistic space-charge-dominated electron bunches for single-shot femtosecond electron diffraction. Phys. Rev. Lett. 105, 264801 (2010) . 21231672 10.1103/PhysRevLett.105.264801

[b65] Waldecker L., Bertoni R. & Ernstorfer R. Compact femtosecond electron diffractometer with 100 keV electron bunches approaching the single-electron pulse duration limit. J. Appl. Phys. 117, 13109–81901 (2015) .

[b66] Gerbig C., Senftleben A., Morgenstern S., Sarpe C. & Baumert T. Spatio-temporal resolution studies on a highly compact ultrafast electron diffractometer. N. J. Phys. 17, 043050 (2015) .

[b67] Sciaini G. *et al.* Electronic acceleration of atomic motions and disordering in bismuth. Nature 458, 56–59 (2009) . 19262668 10.1038/nature07788

[b68] Li R. K., Musumeci P., Bender H. A., Wilcox N. S. & Wu M. Imaging single electrons to enable the generation of ultrashort beams for single-shot femtosecond relativistic electron diffraction. J. Appl. Phys. 110, 074512 (2011) .

[b69] Beye M. *et al.* X-ray pulse preserving single-shot optical cross-correlation method for improved experimental temporal resolution. Appl. Phys. Lett. 100, 1–5 (2012) .

[b70] Battaglia M. *et al.* Characterisation of a CMOS active pixel sensor for use in the TEAM microscope. Nucl. Instruments Methods Phys. Res. Sect. A Accel. Spectrometers Detect. Assoc. Equip. 622, 669–677 (2010) .

